# PW03-014B - Gene-expression profiling study in FMF families

**DOI:** 10.1186/1546-0096-11-S1-A241

**Published:** 2013-11-08

**Authors:** B Aygun, M Carrabba, M Fabbri, L Gribaldo, M Zarantonello, G Fabio

**Affiliations:** 1 European Commission - Joint Research Centre, Institute for Health and Consumer Protection – Molecular Biology and Genomics Unit, Ispra, Italy; 2 Internal Medicine Department, Fondazione IRCCS Ca' Granda Ospedale Maggiore Policlinico, Italy; 3 Medical Science and Community Health Department, Universita' degli Studi, Milan, Italy; 4 Experimental and Clinical Medicine Department, University of Insubria, Varese, Italy

## Introduction

The inflammasome complex and the inflammatory pathway have been implicated in the pathogenesis of familial Mediterranean fever (FMF), and recently ASC (apoptosis-associated speck-like protein containing a CARD) mRNA expression was found to be up-regulated in patients carrying MEFV mutations independent of the mutation type. Gene-expression profiling has the capacity to reveal transcriptome profiles to discriminate disease "phenotypes" to understand a given disease and bring to light new diagnostic and predictive biomarkers.

## Objectives

This pilot study focuses on comparative gene expression profiling in FMF patients with different phenotypes while bearing the same mutations in the search of signature patterns.

## Methods

Members of two different families representing different mutation sets were subjects of the study. mRNA expression in carriers of the same MEFV mutation that exhibit different phenotypes was comparatively analyzed. Total mRNA was extracted from peripheral blood. Microarray analysis was run on Human 8X60 K Microarray slides and analyzed for whole genome expression levels using an Agilent Scanner.

## Results

A within-group analysis of candidate genes for each family was carried out by comparison of gene expression levels in all family members with clinical FMF versus the member without FMF. Although variation in mRNA levels of inflammation related genes such as NLRP3, NLRP1, PYCARD, MEFV, NLRC4, CASP1, PSTPIP1 was observed it was not possible to identify a signature pattern of expression either within-family analysis or comparison between investigated families.

Subsequently, total gene expression was analysed using as threshold a log fold change of 1 or -1 and pathway enrichment was performed using DAVID (the Database for Annotation, Visualization and Integrated Discovery).

In the first family composed of the grandmother, her son and her nephew, all heterozygous for M694V, we compared the two subjects with clinical diagnosis of FMF (son and nephew), in complete remission under colchicine, to the healthy carrier (grandmother).We obtained a list of 73 genes regulated in the same direction. A significant enrichment in the pathways of regulation of amyloid precursor protein biosynthetic process, regulation of T-helper 2 type immune response, regulation of glycoprotein biosynthetic process was found.

In the second family, composed of the father, his son and his daughter all carrying the complex allele E148Q/R761H, we compared the two subjects with clinical diagnosis of FMF (son and daughter), in complete remission under colchicine, to the healthy carrier (father). We obtained a list of 48 genes regulated in the same direction. A significant enrichment was found in the pathways of negative regulation of apoptosis, inflammatory/defense response, cellular ion homeostasis, regulation of activated T cell proliferation.

## Conclusion

This pilot study investigated pathways involved in genotype/phenotype correlation in two families manifesting FMF via mRNA gene-expression profiling. Different mutations seem to exert their effects involving different pathways. For any conclusive generalization higher number of patients and representative families are needed to be investigated.

## Disclosure of interest

None declared

